# Efficacy of the combination of water aerobics and metacognitive training on psychological and physical health variables and their relationship with SP1 and SP4 biomarkers in people with psychosis: a study protocol

**DOI:** 10.3389/fpsyg.2024.1360004

**Published:** 2024-06-11

**Authors:** Susana Ochoa, Marina Verdaguer-Rodríguez, Núria Batlle, Francesc Garreta, Berta Garcia, Josep María Haro, Èlia Vila-Andreu, Maria José Hernández, Maria José Escandell, Ana Muñoz, Sònia Vilamala, Sandra Marcos, Laura Bassolas, María Pascua, Belén Ramos, on behalf of Thalassa Research Group

**Affiliations:** ^1^Etiopatogènia i Tractament dels Trastorns Mentals Greus (MERITT), Institut de Recerca Sant Joan de Déu, Esplugues de Llobregat, Spain; ^2^Parc Sanitari Sant Joan de Déu, España, Spain; ^3^Investigación Biomédica en Red de Salud Mental (CIBERSAM), Madrid, Spain; ^4^Clinical and Health Psychology Department, Psychology Faculty, Universitat Autònoma de Barcelona, Cerdanyola del Vallès, Spain; ^5^Epidemiologia dels trastorns mentals i de l'envelliment, Institut de Recerca Sant Joan de Déu, Esplugues de Llobregat, Spain; ^6^Facultat de Medicina, Departament de Bioquímica i Biologia Molecular, Universitat Autònoma de Barcelona, Cerdanyola del Vallès, Spain; ^7^Faculty of Medicine, University of Vic-Central University of Catalonia, Vic, Spain

**Keywords:** schizophrenia, physical exercise, metacognition, specificity protein, psychological interventions

## Abstract

**Background:**

Metacognitive Training (MCT) is widely used and effective in reducing positive symptoms in psychosis. Physical exercise, such as Water Aerobics (WA), improves general health, quality of life and symptoms as a low impact activity that allows social interactions. Preliminary results suggest a relationship between dopamine and psychotic symptoms, through SP transcription factors, SP1 and SP4 biomarkers. The aims of the project are to evaluate the efficacy of a combined intervention (WA and MCT) for psychosis to improve psychotic symptoms, physical health, and transcription levels of SP biomarkers.

**Materials and methods:**

This is a unicentric randomized controlled trial of three parallel intervention groups: MCT, WA and combined intervention. The estimated sample will be 48 patients with a psychotic spectrum disorder diagnosis. The assessment will be performed at baseline and at 2-months’ follow-up. Instruments used in the assessment will include clinical, cognitive, metacognitive, social cognitive and psychosocial variables.

**Discussion:**

This will be the first study investigating the impact of the combination of MCT and WA in psychosis. Moreover, it will be the first study analyzing changes in the transcriptional biomarkers SP1 and SP4 after interventions. The results of this study may have clinical implications contributing to the improvement of treatment selection.

**Clinical trial registration:**

https://clinicaltrials.gov/, identifier: NCT05455593.

## 1 Introduction

Schizophrenia and other psychotic spectrum disorders are severe mental disorders that affect around 1% of the population (Ochoa et al., 2008). Positive symptoms such as delusions and hallucinations usually respond to medication. Nevertheless, psychotic symptoms are sometimes resistant to antipsychotic medication, which is often not effective enough to achieve functional recovery (Morrison et al., 2018).

In this context, psychological treatment can help improve symptoms and functioning, even in the absence of pharmacological treatment (Morrison et al., 2014). Treatments such as Metacognitive Training (MCT) (Moritz and Woodward, 2007) have shown solid evidence for the improvement of psychotic symptoms. MCT is based on Cognitive-Behavioral Therapy and Psychoeducation, and it was developed to address cognitive biases and social cognition deficits in patients with psychosis. The focus of MCT is promoting reflectiveness on thought processes. It is widely used and effective in reducing positive symptoms, improving cognitive functioning, cognitive insight, immediate memory, and quality of life in schizophrenia (Moritz et al., 2014; Pankowski et al., 2016). Those improvements have already been established as possible in early stages of the disorder (Ochoa et al., 2017; Ruiz-Delgado et al., 2022). Moreover, previous studies have found that MCT can lead to immediate and sustained improvements in symptoms for individuals with psychosis (Moritz et al., 2014; Penney et al., 2022). In addition, evidence shows that MCT can be effective for both men and women with first-episode psychosis but target distinct aspects. Salas-Sender et al. (2020) found that after MCT, women with first-episode psychosis (FEP) experienced improvements in general symptoms, cognitive insight, and attributional style, while men showed a reduced presence of the jumping to conclusion bias.

Schizophrenia also has negative effects on general health and quality of life. In this line, physical exercise represents a promising new treatment option to complement current psychosocial and pharmacological interventions for psychosis. Evidence suggests that physical exercise can improve cardio-metabolic functioning and facilitate neurogenesis in brain areas markedly affected in psychosis (Gurusamy et al., 2018). It should be noted that metabolic syndrome is very common in patients with severe mental illnesses, seriously affecting their physical health, so physical exercise would have a beneficial impact on reducing this issue (Cortés, 2011). Regarding mental health, a recent review by Mittal et al. (2017) found that moderate-intensity physical exercise can be beneficial for improving psychotic symptoms, both positive and negative, cognition, psychosocial functioning, social stigma and motor coordination, in addition to weight loss and BMI reduction. Most previous studies were based on the implementation of aerobic-type exercises, although other exercises involving motor coordination may have similar results (Firth et al., 2016). In this line, Water Aerobics (WA) has an impact in motor coordination, and previous studies indicate that it has been effective in reducing anxiety and depression in pre-and postpartum women (Davenport et al., 2018; Mottola et al., 2018). Additionally, water immersion contributes to improving health in several ways: from cardiovascular function to skeleto-muscular pain (An et al., 2019) and fostering social relationships in a rewarding environment (Vázquez Lara et al., 2017). WA can be a promising therapy for improving the mental health of individuals with schizophrenia, as other types of physical exercise have demonstrated an improvement in cognition, clinical symptoms, and quality of life in patients with schizophrenia. While running and weight training are also beneficial for people living with schizophrenia, swimming is a low-impact activity that may be more accessible to some individuals (Firth et al., 2016; Midtgaard et al., 2021).

In the context of brain-level alterations, positive symptoms of psychosis are associated with dopamine (DA) in subcortical areas of the brain (Toda and Abi-Dargham, 2007), and with the glutamatergic pathway (Jentsch, 1999). The expression of these components is regulated by specificity protein (SP) transcription factors (Priya et al., 2013). It has been described that SP transcription factors are altered in the brain tissue of people with psychotic disorders (Pinacho et al., 2011, 2013). In FEP, reduced levels of SP1 and SP4 biomarkers have been found in circulating lymphocytes. Specifically, SP4 has been associated with improvements in symptoms in women with schizophrenia treated with raloxifene (Vila et al., 2019). Additionally, cognitive deficits have been detected in animals with reduced SP4 levels (Zhou et al., 2010). SP4 is involved in the development of dendritic trees in brain and hippocampal granule neurons (Ramos et al., 2007; Zhou et al., 2010), suggesting that SP4 might be linked to schizophrenia in the context of the theory of neurodevelopment (Zhou et al., 2010). Moreover, some studies suggest that SP4 would not only act as a high-risk gene for psychosis. Instead, it could contribute to the regulation network of other genes related to the risk of developing psychosis (Zhou, 2022). Recent literature has identified a clear relationship between the cerebellum and the prefrontal cortex via the cortico-cerebellum-thalamic-cortical circuitry, both in mice and in people with schizophrenia. Evidence shows that stimulation of the signal return from the cerebellum to the thalamus would generate improvements in psychotic symptoms (Parker et al., 2017; Brady et al., 2019; Escelsior and Belvederi, 2019). In addition, it has recently been identified that the cerebellum has dopaminergic projections toward the ventral tegmental area that would ultimately modulate the prefrontal cortex (Carta et al., 2019). This study, together with previous findings showing that SP4 and SP1 control the expression of D2 receptor in the cerebellum in schizophrenia (Pinacho et al., 2013), suggests a possible role of the transcription factors SP1 and SP4 in this circuit from the cerebellum to prefrontal cortex. Considering these pathways, it would be interesting to study the effect of an intervention that integrates stimulation of the cerebellum and cortex at the same time. The cerebellum is the area in charge of motor coordination through its connection with the motor cortex, with pathways to the prefrontal cortex that relay in the thalamus or ventral tegmental area. We expect that the stimulation of the cerebellum through motor coordination tasks such as WA could influence prefrontal cortex activity and therefore cognitive functions dependent on this area. On the other hand, MCT is an intervention aiming to improve self-reflectiveness and metacognition, which are functions associated with the prefrontal area of the brain. Therefore, we believe that the application of these interventions will exponentially improve symptoms, cognitive and metacognitive functioning, and social functionality, while also assuming an improvement in the quality of life of people with psychosis.

On one hand, we expect that WA intervention will improve physical health variables. On the other hand, we expect that MCT will improve variables related to symptoms and metacognition. Therefore, our main hypothesis is that the combined intervention will enhance the benefits of WA and MCT separately. The project will aim to evaluate the effects of each intervention, comparing the changes in the explored variables between the 3 intervention groups. Specifically, it is expected that the combined intervention will greatly facilitate an improvement in symptoms, cognition, metacognition, and psychosocial functioning. In addition, the transcript levels of the SP1 and SP4 biomarkers may change after the interventions, and it is anticipated that these changes will be even greater when the two interventions are combined.

The objectives of this project are:

1. To evaluate the efficacy of a combined intervention (WA and MCT) in people with schizophrenia or other psychotic disorders compared to each of the independent interventions on:
  - Psychotic symptoms, cognition, metacognition, psychosocial functioning, and social stigma
  - Motor coordination
  - State of physical health: weight, height, BMI, abdominal circumference, blood pressure, heart rate, analysis that includes information on glucose, total cholesterol, HDL, and LDL
2. Study changes in the transcription levels of the SP1 and SP4 biomarkers depending on the intervention received (WA, MCT or combined intervention).
3. Associate changes in SP1 and SP4 levels to improvements in symptoms, cognition, metacognition, social functioning, social stigma, motor coordination, and physical health status.

## 2 Materials and methods

### 2.1 Study design

This is a unicentric randomized controlled trial with 3 parallel intervention groups. Participating services will recruit patients, and they will be randomized to each condition. Following baseline assessment, a random number table is used to assign each patient to one of the three conditions.

Conditions are (1) MCT, (2) WA, and (3) Combined Intervention (MCT and WA). The interventions include 8 weekly sessions, lasting approximately 2 months. We will perform post-treatment assessments right after the treatment ends. Upon completion of the last assessment, patients will be offered the opportunity to participate in any of the activities, regardless of the group assigned during the 2-month intervention. Details on the flowchart of the study can be seen in Supplementary Figure S1.

### 2.2 Participants

The sample consists of patients with a diagnosis of psychotic disorder, according to DSM-5 criteria (American Psychiatric Association, 2014). Inpatients and outpatients are recruited from Parc Sanitari Sant Joan de Déu Mental Health Network. Inclusion criteria are (1) diagnosis of schizophrenia, schizoaffective disorder, delusional disorder, brief psychotic disorder, schizophreniform disorder or unspecific psychotic disorder; (2) Psychopathological stability; (3) age between 18 and 60 years old. Exclusion criteria are (1) head trauma, dementia or intellectual disability (premorbid IQ ≤ 70); (2) PANSS scores of ≥5 in hostility, lack of cooperation or suspiciousness; (3) diagnosis of substance dependence disorder; (4) limitations to exercise in water, such as severe mobility problems or specific phobias related to water.

### 2.3 Measures

We will collect data about clinical, cognitive, metacognitive, social cognitive and psychosocial variables. We will include a sociodemographic questionnaire. Data on antipsychotic treatment will be transformed into chlorpromazine defined daily dose. The instruments used for the assessment of clinical, cognitive, metacognitive, social cognitive and psychosocial variables are reported in Supplementary Table S1.

Molecular measures of SP1 and SP4 biomarkers will be gathered. Specifically, protein transcription factor levels and mRNA expression levels in peripheral blood mononuclear cells (PBMCs) will be obtained. Moreover, somatometric data weight (kilograms), height (meters), body mass index (BMI, in kg/m^2), body abdominal girth (centimeters) and blood pressure (mmHg). We will also measure heart rate (bpm) and metabolic parameters such as glucose levels (mg/dL), HDL, LDL and total cholesterol levels (mg/dL).

### 2.4 Data collection

A trained psychologist will perform baseline and post-treatment assessments of sociodemographic, clinical, functioning, metacognitive and social cognitive variables. Each assessment will consist of 2–3 interviews. Nursing staff will take anatomical measures and conduct hemograms from the cubital vein for the separation of peripheral blood mononuclear cells. The blood will be processed by the molecular psychiatry laboratory on the same day as the hemogram and will be stored at −80°C until molecular analysis.

This study is in accordance with the World Medical Association’s Declaration of Helsinki and was approved by the Sant Joan de Déu Ethics Committee (Reference: PIC-155-21). The study will involve human participants and we will obtain informed consent prior to the start of the assessments. The staff performing the assessments, hemograms and molecular analyses will be blind to the study condition assigned to the participants. All variables will be measured at baseline and after the 2-month intervention, except the WAIS-III Vocabulary subscale, which will only be administered at baseline. After the 2-month intervention, we will collect qualitative data on patient satisfaction with the intervention, based on a questionnaire created *ad hoc*.

### 2.5 Interventions

Participants who meet inclusion criteria will be randomized to one of 3 study conditions: (1) Metacognitive Training (MCT), (2) Water Aerobics (WA), and (3) Combined Intervention (MCT and WA).

Metacognitive Training (MCT): MCT consists of 8 group sessions of 1 h each and weekly frequency. Different metacognitive aspects related to the most common biases that occur in people with schizophrenia are worked on. The sessions are: attributional style, jumping to conclusions (2 sessions), changing beliefs, theory of mind and emotional recognition (2 sessions), memory errors and depression and mood. The groups are structured, include visual material, are entertaining and very participative and include discussions on how misinterpreting some situations can lead to exaggerated responses. Supplementary Table S2 shows each of the sessions of the MCT intervention.

Water Aerobics (WA): each weekly session will be 45–60 min in duration. Each activity (WA and MCT session) will take place once a week. Therefore, participants assigned to the combined intervention condition will attend both activities each week.

Participants assigned to the MCT condition will be offered WA after the completion of the study. Participants assigned to the WA condition will be offered to participate in a MCT group after the completion of the study. Participants assigned to the combined intervention condition will be offered the chance to continue participating in WA after the completion of the study, if interested.

An intervention guide will be developed in which Water Aerobics will be promoted. This guide will be elaborated by professional experts in physical activity science, physiotherapy, medicine, and sports psychology.

### 2.6 Sample size calculation

Based on previous results obtained in a MCT efficacy clinical trial (Ochoa et al., 2017) and accepting an alpha risk of 0.05 and a beta risk of less than 0.2 in a bilateral contrast, we would need 48 subjects to detect a difference equal to or greater than 1.7 units in the BCIS metacognitive variable, assuming a SD of 3.63. A loss to follow-up of 25% has been estimated.

### 2.7 Data analysis

Repeated measures models will be performed comparing the results of the three groups according to the study variables, clinical, cognitive, metacognitive, and social functioning, social stigma, motor coordination, physical health status, as well as biomarker variables. We will include as covariates those variables that show significant mean differences between groups in the baseline assessment. Additionally, we will control by sex, age, antipsychotic dose (chlorpromazine levels) and symptom severity, since these are variables that have been associated with our main outcomes.

## 3 Expected results and discussion

To the best of our knowledge, this is the first study to investigate the effectiveness of a combination of MCT and WA in a sample of people with psychosis. Additionally, this is the first study that evaluates changes in the transcriptional biomarkers SP1 and SP4 after the implementation of these interventions.

If the hypotheses of this study are confirmed, they have dual applicability. On the one hand, the evidence shows how MCT improves psychotic symptoms, especially positive symptoms, cognitive insight, cognition, and cognitive biases (Penney et al., 2022). On the other hand, physical exercise can improve psychotic symptoms, neurocognition, social functioning, social stigma, motor coordination and several physical health parameters (Firth et al., 2018). Specifically, WA would be a promising option for the comprehensive treatment of psychosis. We hypothesize that the combination of both interventions can optimize the benefits that each has separately. Also, the combination of MCT and WA could influence changes in biomarkers related to psychosis. This would reflect the importance of considering gene reprogramming for the treatment to be effective. Therefore, this article would support the need to combine psychological interventions and physical exercise to improve mental health.

Most researchers and clinicians agree that the treatment of patients with psychosis should be personalized and adapted to their specific needs (Package of Interventions for Rehabilitation, 2023). However, this is seldom done in clinical practice. One of the reasons is the economic cost of tailored treatments. Another important reason is the lack of scientific knowledge on the grounds that should guide the personalization of interventions. This is a current challenge in research in mental health. MCT is a perfect candidate to study its viability as a personalized treatment for several reasons. Firstly, it has proven its short-and long-term efficacy (Moritz et al., 2014; Ochoa et al., 2017; Penney et al., 2022) and it is currently included as a treatment of choice for people with psychosis in therapeutic guidelines and targets the psychological foundations of psychosis (Package of Interventions for Rehabilitation, 2023). Secondly, it has been studied in different subpopulations of psychosis showing positive results in different diagnoses and stages of the illness. Additionally, different profiles of patients could benefit from MCT considering their baseline assessment (Ferrer-Quintero et al., 2021, 2022). Thirdly, it is standardized through a manual guide, that allows comparison between different hospitals and facilitates delivery by any mental health professional with adequate training. Finally, the intervention is conducted in a group setting, thus being more cost-effective than individual treatment. The combination of MCT and WA could be a future area of interest in the personalization of treatment for people with psychosis. Additionally, Firth et al. (2018) found that once physical exercise is not offered by the mental health services, the adherence to the activity diminished by half. So, in the future, tailored interventions should also consider the inclusion of these activities in their service portfolio.

One limitation of the study is the absence of reevaluation of the measures long term. Due to limited resources, the study only offers measures post-intervention, that mark the effectiveness of the combined training. Nevertheless, we expect that the benefits would persist in the long-term considering previous MCT studies (Moritz et al., 2014). In the same way, patients in this study are selected from different mental health services, in different stages of the disorder, with different severity impact. This could impact the clinical response, due to heterogeneity. Nevertheless, this heterogeneity could contribute positively, allowing the implementation of the interventions in several settings and stages. Another limitation could be that we have not considered tobacco dependence as an exclusion criteria, due to its possible effect on cognition and symptoms. We have not excluded tobacco dependence because of the high prevalence of patients with schizophrenia that consume it. Including this as an exclusion criteria would severely limit the possibility of recruitment, and it would reduce ecological validity. However, it would be interesting if further studies consider this variable, due to its possible effect in cognitive function (Núñez et al., 2015; Hickling et al., 2018; Ding and Hu, 2021).

Further studies should consider the profile of response to each of these two interventions, or its combination, in order to design a more personalized approach in their treatment, based on stages of the illness and the patients’ needs.

In summary, these results could be of great importance for the understanding, prevention and treatment of schizophrenia and other psychotic disorders.

## Ethics statement

The studies involving humans were approved by the Sant Joan de Déu Ethics Committee (Reference: PIC-155-21). The studies were conducted in accordance with the local legislation and institutional requirements. Written informed consent will be obtained from the participants prior to the start of the assessments.

## Author contributions

SO: Conceptualization, Funding acquisition, Methodology, Project administration, Resources, Supervision, Visualization, Writing – original draft, Writing – review & editing. MV-R: Conceptualization, Methodology, Project administration, Resources, Visualization, Writing – original draft, Writing – review & editing. NB: Resources, Writing – original draft, Writing – review & editing. FG: Conceptualization, Funding acquisition, Writing – original draft. BG: Conceptualization, Resources, Validation, Writing – original draft. JH: Conceptualization, Funding acquisition, Methodology, Writing – original draft. ÈV-A: Conceptualization, Resources, Writing – original draft. MH: Resources, Writing – review & editing. ME: Resources, Writing – review & editing. AM: Resources, Writing – review & editing. SV: Resources, Writing – review & editing. SM: Resources, Writing – review & editing. LB: Resources, Writing – review & editing. MP: Resources, Writing – review & editing. BR: Conceptualization, Methodology, Resources, Supervision, Writing – original draft.

## Thalassa Research Group

Juan Ramon Albalate, Laura Altimira, Jordi Antolín, Belén Arranz, Laura Bassolas, Núria Batlle, Irene Birulés, Jose Luis Bogas, Eva Carrasco, Gloria Comellas, Montserrat Contel, Marta Domínguez, Maria José Escandell, Ana Escanilla, Eva Ferrer, Berta Garcia, Francesc Garreta, Laura González, Maria José Hernández, Marcos Hidalgo, Antonia Manrique, Sandra Marcos, Laura Marin, Irene Martínez, Bienvenido Mateo, Cristina Montejo, Ana Muñoz, Laura Nicolau, Marta Núñez, Susana Ochoa, María Pascua, Laura Prats, Belén Ramos, Belén Ribas, Gemma Riquelme, Carlota Romans, David Romero, Rosana Romero, Maria Inmaculada Ruiz, Helena Sainz, Elisabet Salomon, Malak Tahri, Marina Vall, Emma Ventura, Marina Verdaguer-Rodríguez, Francisco Vidal, Èlia Vila-Andreu, Sònia Vilamala, Míriam Vilaplana, Josep María Haro.

## References

[ref1] American Psychiatric Association (2014). Guía de consulta de los criterios diagnósticos del DSM-5. Arlington, VA: American Psychiatric Publishing, 438.

[ref2] An J. Lee I. Yi Y. (2019). The thermal effects of water immersion on health outcomes: An integrative review. IJERPH 16:1280. doi: 10.3390/ijerph16071280 30974799 PMC6479732

[ref3] Brady R. O. Gonsalvez I. Lee I. Öngür D. Seidman L. J. Schmahmann J. D. . (2019). Cerebellar-prefrontal network connectivity and negative symptoms in schizophrenia. AJP 176, 512–520. doi: 10.1176/appi.ajp.2018.18040429, PMID: 30696271 PMC6760327

[ref4] Carta I. Chen C. H. Schott A. L. Dorizan S. Khodakhah K. (2019). Cerebellar modulation of the reward circuitry and social behavior. Science 363:eaav0581. doi: 10.1126/science.aav0581, PMID: 30655412 PMC6711161

[ref5] Cortés M. B. (2011). Síndrome metabólico y antipsicóticos de segunda generación. Rev Asoc Esp Neuropsiq. 31, 303–320. doi: 10.4321/S0211-57352011000200009

[ref6] Davenport M. H. McCurdy A. P. Mottola M. F. Skow R. J. Meah V. L. Poitras V. J. . (2018). Impact of prenatal exercise on both prenatal and postnatal anxiety and depressive symptoms: a systematic review and meta-analysis. Br. J. Sports Med. 52, 1376–1385. doi: 10.1136/bjsports-2018-099697, PMID: 30337464

[ref9002] Ding J. B. Hu K. (2021). Cigarette Smoking and Schizophrenia: Etiology, Clinical, Pharmacological, and Treatment Implications. Schizophrenia research and treatment. 2021:7698030. doi: 10.1155/2021/7698030 34938579 PMC8687814

[ref7] Escelsior A. Belvederi M. M. (2019). Modulation of cerebellar activity in schizophrenia: is it the time for clinical trials? Schizophr. Bull. 45, 947–949. doi: 10.1093/schbul/sbz017, PMID: 30932162 PMC6737480

[ref8] Ferrer-Quintero M. Fernández D. López-Carrilero R. Birulés I. Barajas A. Lorente-Rovira E. . (2021). Persons with first episode psychosis have distinct profiles of social cognition and metacognition. NPJ Schizophr. 7:61. doi: 10.1038/s41537-021-00187-8, PMID: 34887442 PMC8660816

[ref9] Ferrer-Quintero M. Fernández D. López-Carrilero R. Birulés I. Barajas A. Lorente-Rovira E. . (2022). Males and females with first episode psychosis present distinct profiles of social cognition and metacognition. Eur. Arch. Psychiatry Clin. Neurosci. 272, 1169–1181. doi: 10.1007/s00406-022-01438-0, PMID: 35802165 PMC9508015

[ref10] Firth J. Carney R. French P. Elliott R. Cotter J. Yung A. R. (2018). Long-term maintenance and effects of exercise in early psychosis: maintaining exercise in early psychosis. Early Interv. Psychiatry 12, 578–585. doi: 10.1111/eip.12365, PMID: 27587302 PMC6099223

[ref11] Firth J. Carney R. Jerome L. Elliott R. French P. Yung A. R. (2016). The effects and determinants of exercise participation in first-episode psychosis: a qualitative study. BMC Psychiatry 16:36. doi: 10.1186/s12888-016-0751-7, PMID: 26896958 PMC4761413

[ref12] Gurusamy J. Gandhi S. Damodharan D. Ganesan V. Palaniappan M. (2018). Exercise, diet and educational interventions for metabolic syndrome in persons with schizophrenia: a systematic review. Asian J. Psychiatr. 36, 73–85. doi: 10.1016/j.ajp.2018.06.018, PMID: 29990631

[ref9003] Hickling L. M. Perez-Iglesias R. Ortiz-García de la Foz V. Balanzá-Martínez V. McGuire P. . (2018). Tobacco smoking and its association with cognition in first episode psychosis patients. Schizophr. Res. 192, 269–273. doi: 10.1016/j.schres.2017.04.018 28412088

[ref13] Jentsch J. (1999). The Neuropsychopharmacology of phencyclidine from NMDA receptor hypofunction to the dopamine hypothesis of schizophrenia. Neuropsychopharmacology 20, 201–225. doi: 10.1016/S0893-133X(98)00060-8, PMID: 10063482

[ref14] Midtgaard J. Schnor H. Bjerre E. D. Jespersen T. Jelsøe N. Frølund N. . (2021). Exercise training complementary to specialised early intervention in patients with first-episode psychosis: a feasibility randomised trial. Pilot Feasibility Stud. 7:162. doi: 10.1186/s40814-021-00900-5, PMID: 34412705 PMC8375206

[ref15] Mittal V. A. Vargas T. Juston Osborne K. Dean D. Gupta T. Ristanovic I. . (2017). Exercise treatments for psychosis: a review. Curr. Treat Options Psych. 4, 152–166. doi: 10.1007/s40501-017-0112-2, PMID: 29034144 PMC5636011

[ref16] Moritz S. Andreou C. Schneider B. C. Wittekind C. E. Menon M. Balzan R. P. . (2014). Sowing the seeds of doubt: a narrative review on metacognitive training in schizophrenia. Clin. Psychol. Rev. 34, 358–366. doi: 10.1016/j.cpr.2014.04.004, PMID: 24866025

[ref17] Moritz S. Veckenstedt R. Andreou C. Bohn F. Hottenrott B. Leighton L. . (2014). Sustained and “sleeper” effects of Group metacognitive training for schizophrenia: a randomized clinical trial. JAMA Psychiatry 71, 1103–1111. doi: 10.1001/jamapsychiatry.2014.1038, PMID: 25103718

[ref18] Moritz S. Woodward T. S. (2007). Metacognitive training in schizophrenia: from basic research to knowledge translation and intervention. Curr. Opin. Psychiatry 20, 619–625. doi: 10.1097/YCO.0b013e3282f0b8ed 17921766

[ref19] Morrison A. P. Pyle M. Gumley A. Schwannauer M. Turkington D. MacLennan G. . (2018). Cognitive behavioural therapy in clozapine-resistant schizophrenia (FOCUS): an assessor-blinded, randomised controlled trial. Lancet Psychiatry 5, 633–643. doi: 10.1016/S2215-0366(18)30184-6, PMID: 30001930 PMC6063993

[ref20] Morrison A. P. Turkington D. Pyle M. Spencer H. Brabban A. Dunn G. . (2014). Cognitive therapy for people with schizophrenia spectrum disorders not taking antipsychotic drugs: a single-blind randomised controlled trial. Lancet 383, 1395–1403. doi: 10.1016/S0140-6736(13)62246-1 24508320

[ref21] Mottola M. F. Davenport M. H. Ruchat S. M. Davies G. A. Poitras V. J. Gray C. E. . (2018). 2019 Canadian guideline for physical activity throughout pregnancy. Br. J. Sports Med. 52, 1339–1346. doi: 10.1136/bjsports-2018-100056, PMID: 30337460

[ref9001] Núñez C. Stephan-Otto C. Cuevas-Esteban J. Maria Haro J. Huerta-Ramos E. Ochoa S. . (2015). Effects of caffeine intake and smoking on neurocognition in schizophrenia. Psychiatry Res. 230, 924–931. doi: 10.1016/j.psychres.2015.11.022 26614014

[ref22] Ochoa S. Haro J. M. Torres J. V. Pinto-Meza A. Palacín C. Bernal M. . (2008). What is the relative importance of self reported psychotic symptoms in epidemiological studies? Results from the ESEMeD—Catalonia Study. Schizophr. Res. 102, 261–269. doi: 10.1016/j.schres.2008.04.010, PMID: 18495432

[ref23] Ochoa S. López-Carrilero R. Barrigón M. L. Pousa E. Barajas A. Lorente-Rovira E. . (2017). Randomized control trial to assess the efficacy of metacognitive training compared with a psycho-educational group in people with a recent-onset psychosis. Psychol. Med. 47, 1573–1584. doi: 10.1017/S0033291716003421, PMID: 28166848

[ref24] Package of Interventions for Rehabilitation (2023). Module 8. Mental health conditions. Geneva: World Health Organization.

[ref25] Pankowski D. Kowalski J. Gawęda Ł. (2016). The effectiveness of metacognitive training for patients with schizophrenia: a narrative systematic review of studies published between 2009 and 2015. Psychiatr. Pol. 50, 787–803. doi: 10.12740/PP/59113, PMID: 27847929

[ref26] Parker K. L. Kim Y. C. Kelley R. M. Nessler A. J. Chen K. H. Muller-Ewald V. A. . (2017). Delta-frequency stimulation of cerebellar projections can compensate for schizophrenia-related medial frontal dysfunction. Mol. Psychiatry 22, 647–655. doi: 10.1038/mp.2017.50, PMID: 28348382 PMC5873945

[ref27] Penney D. Sauvé G. Mendelson D. Thibaudeau É. Moritz S. Lepage M. (2022). Immediate and sustained outcomes and moderators associated with metacognitive training for psychosis: a systematic review and Meta-analysis. JAMA Psychiatry 79, 417–429. doi: 10.1001/jamapsychiatry.2022.0277, PMID: 35320347 PMC8943641

[ref28] Pinacho R. Villalmanzo N. Lalonde J. Haro J. M. Meana J. J. Gill G. . (2011). The transcription factor SP4 is reduced in postmortem cerebellum of bipolar disorder subjects: control by depolarization and lithium. Bipolar Disord. 13, 474–485. doi: 10.1111/j.1399-5618.2011.00941.x, PMID: 22017217 PMC3202296

[ref29] Pinacho R. Villalmanzo N. Roca M. Iniesta R. Monje A. Haro J. M. . (2013). Analysis of Sp transcription factors in the postmortem brain of chronic schizophrenia: a pilot study of relationship to negative symptoms. J. Psychiatr. Res. 47, 926–934. doi: 10.1016/j.jpsychires.2013.03.004, PMID: 23540600

[ref30] Priya A. Johar K. Wong-Riley M. T. T. (2013). Specificity protein 4 functionally regulates the transcription of NMDA receptor subunits Glu N1, GluN2A, and GluN2B. Biochimica et Biophysica Acta (BBA) - molecular. Cell Res. 1833, 2745–2756. doi: 10.1016/j.bbamcr.2013.07.002 PMC3834217 23871830

[ref31] Ramos B. Gaudillière B. Bonni A. Gill G. (2007). Transcription factor Sp4 regulates dendritic patterning during cerebellar maturation. Proc. Natl. Acad. Sci. USA 104, 9882–9887. doi: 10.1073/pnas.0701946104, PMID: 17535924 PMC1887555

[ref32] Ruiz-Delgado I. Moreno-Küstner B. García-Medina M. Barrigón M. L. Gonzalez-Higueras F. López-Carrilero R. . (2022). Is metacognitive training effective for improving neurocognitive function in patients with a recent onset of psychosis? Psychiatry Res. 318:114941. doi: 10.1016/j.psychres.2022.114941, PMID: 36375331

[ref33] Salas-Sender M. López-Carrilero R. Barajas A. Lorente-Rovira E. Pousa E. Barrigón M. L. . (2020). Gender differences in response to metacognitive training in people with first-episode psychosis. J. Consult. Clin. Psychol. 88, 516–525. doi: 10.1037/ccp0000468, PMID: 31855037

[ref34] Toda M. Abi-Dargham A. (2007). Dopamine hypothesis of schizophrenia: making sense of it all. Curr. Psychiatry Rep. 9, 329–336. doi: 10.1007/s11920-007-0041-7, PMID: 17880866

[ref35] Vázquez Lara J. Díaz L. R. Rodrigo J. R. Gutiérrez V. Luque G. T. Gómez-Salgado J. (2017). Calidad De Vida Relacionada Con La Salud En Una Población De Gestantes Sanas Tras Un Programa De Actividad Física En El Medio Acuático (Pafmae). Rev Esp Salud Pública. 91:e201710042. Available at: http://scielo.isciii.es/scielo.php?script=sci_arttext&pid=S1135-57272017000100419&lng=es&tlng=es. 29083398 PMC11587378

[ref36] Vila È. Huerta-Ramos E. Núñez C. Usall J. Ramos B. (2019). Specificity proteins 1 and 4 in peripheral blood mononuclear cells in postmenopausal women with schizophrenia: a 24-week double-blind, randomized, parallel, placebo-controlled trial. Eur. Arch. Psychiatry Clin. Neurosci. 269, 941–948. doi: 10.1007/s00406-018-0938-7, PMID: 30167782

[ref37] Zhou X. (2022). Over-representation of potential SP4 target genes within schizophrenia-risk genes. Mol. Psychiatry 27, 849–854. doi: 10.1038/s41380-021-01376-8, PMID: 34750502 PMC9054665

[ref38] Zhou X. Nie Z. Roberts A. Zhang D. Sebat J. Malhotra D. . (2010). Reduced NMDAR1 expression in the Sp4 hypomorphic mouse may contribute to endophenotypes of human psychiatric disorders. Hum. Mol. Genet. 19, 3797–3805. doi: 10.1093/hmg/ddq298, PMID: 20634195 PMC2935857

