# Supplementary material for: Efficacy of the combination of water aerobics and metacognitive training on psychological and physical health variables and their relationship with SP1 and SP4 biomarkers in people with psychosis: a study protocol
Source: Front Psychol. 2024 Jun 11;15:1360004. doi: 10.3389/fpsyg.2024.1360004 (PMC11197846; doi:10.3389/fpsyg.2024.1360004)

Supplementary Material

# Tables and figures

Table 1 - Instruments

| **INSTRUMENT** | **OUTCOME** | **RESPONSE RANGE** |
| --- | --- | --- |
| **Clinical variables** | | |
| **PANSS: Positive and Negative Syndrome Scale**  *Kay, Fizbein, and Opler, 1987; Peralta and Cuesta, 1994* | Psychotic symptoms | Positive and negative symptoms scales: scores 7 to 49  General symptoms subscale: scores 16 to 112 |
| **BDI: Beck Depression Inventory**  *Beck, 1978; Sanz and Vázquez, 1998* | Depressive symptoms | Higher scores indicate greater severity of symptoms: scores 0 to 63) |
| **RSES: Rosenberg Self-Esteem Scale**  *Rosenberg, 1989; Martín-Albo, Núñez, Navarro, and Grijalvo, 2007* | Self-Esteem | Higher scores indicate better self-esteem: scores 10 to 40 |
| **SUMD: Scale to Assess Unawareness in Mental Disorders**  *Amador et al., 1993; Ruiz et al., 2008* | Clinical insight | Higher scores indicate more unawareness: scores 3 to 15 |
| **Social cognition** | | |
| **IPSAQ: Internal, Personal and Situational Attributions Questionnaire**  *Kinderman and Bentall, 1989; Díez-Alegría and Vázquez-Valverde, 2006* | Attributional style | Externalizing Bias: positive scores indicate a stronger bias.  Personalizing Bias: scores higher than 0.5 indicate a higher proportion of external personal attributions for negative events, compared to situational attributions. |
| **Hinting Task**  *Corcoran, Mercer, and Frith, 1995; Gil, Fernández-Modamio, Bengochea, and Arrieta, 2012* | Theory of mind | Lower scores indicate worse performance: scores 0 to 12 |
| **Faces Test**  *Baron-Cohen, Wheelwright, and Jolliffe, 1997; Huerta-Ramos et al., 2021* | Emotion Processing | Higher scores indicate better emotional processing performance: scores 0 to 20 |
| **Metacognition** | | |
| **BCIS: Beck Cognitive Insight Scale**  *Beck, Baruch, and Balter, 2004; Gutiérrez-Zotes, Valero, and Escartin, 2012* | Cognitive insight | Self-reflectivity, higher scores indicate a greater tendency to question one's beliefs: scores 0 to 27.  Self-certainty, higher scores indicate greater certainty in one's interpretations: scores 0 to 18.  Composite index: scores -27 to 45. |
| **Beads Task**  *Brett-Jones, Garety, and Hemsley, 1987* | Jumping to conclusions (JTC) bias | Draws to decision: scores 0-2 indicate presence of JTC bias, scores above 2 indicate absence of JTC bias |
| **CBQ : Cognitive Biases Questionnaire for Psychosis**  *Peters et al., 2013; Gutiérrez-Zotes, 2021* | Cognitive biases in psychosis | Higher scores indicate greater presence of cognitive biases: scores 30 to 90. |
| **Cognition** | | |
| **MCCB: MATRICS Consensus Cognitive Battery (MSCEIT will not be administered)**  *Nuechterlein et al., 2008; Rodriguez-Jimenez et al., 2012* | 6 domains: processing speed, vigilance, working memory, verbal learning, visual learning, reasoning and problem solving. | TMT A, TMT B, NAB: higher scores indicate worse performance  BACS-SC, HVLT-R, WMS-III, LNS, BVMT-R, Fluency, CPT: higher scores indicate better performance |
| **WAIS-III: Vocabulary subscale, Wechsler Adults Intelligence Scale**  *Wechsler, 1999; Gonzalez-Blanch, 2011* | Premorbid intelligence | Higher scores indicate higher premorbid intelligence: direct scores 0 to 66 |
| **CBQ : Cognitive Biases Questionnaire for Psychosis**  *Peters et al., 2013; Gutiérrez-Zotes, 2021* | Cognitive biases in psychosis | Higher scores indicate greater presence of cognitive biases: scores 30 to 90 |
| **Psychosocial functioning** | | |
| **GAF: Global Assessment of Functioning**  *Endicott, Spitzer, Fleiss, and Cohen, 1976; Bobes, García-Portilla, Bascarán, Sáiz, and Bousoño, 2002* | General functioning | Higher scores indicate better general functioning (scores 0 to 100) |
| **WHODAS 2.0: WHO Disability Assessment Schedule 2.0**  *World Health Organization, 2010* | Health and disability | Higher scores indicate higher levels of disability |
| **Social stigma** | | |
| **SSQ : Self-Stigma Questionnaire**  *Ochoa, 2015* | Self-stigma | Higher scores indicate a higher perception of self-stigma: scores 14-98 |
| **Quality of life** | | |
| **EQ-5D EuroQoL**  *Badia et al., 1999* | Quality of life | 3-point scale ranging from absence, moderate, and extreme health problems, or difficulties. General health status score: higher score indicates a better perception of general health status: score 0 - 100 |
| **IPAQ : International Physical Activity Questionnaire**  *Booth, 2000; Carrera, 2017* | Intensity of physical activity and sitting time | Physical activities: vigorous, moderate, and walking, measured in days per week and minutes per day (higher scores indicate higher level of physical activity).  Sitting time: measured in minutes per day (higher scores indicate higher level of sedentarism) |
| **OSQ : Oviedo Sleep Questionnaire**  *Bobes, 2000* | Sleep quality | Different response ranges throughout 11 items |

# Table 2 – Metacognitive Training sessions

| **STRUCTURE OF THE SESSIONS** | 1. Introduction: Explanation of metacognition and the aim of the module. 2. Presentation of the content and explanation of the different cognitive exercises. 3. Realization of the different exercises. 4. End of the session: Application of the concept to daily life, summary of objectives, training and tasks |
| --- | --- |
| **SESSION 1:**  **ACCUSE AND TAKE CREDIT** | Focus: External attributional style for negative events. Monocausal inferences: only one explanation considered to understand the cause of a complex event.  Aim: To point out that multiple factors can contribute to an event or situation. |
| **SESSION 2:**  **JUMPING TO CONCLUSIONS** | Focus: Jumping to conclusions bias.  Aim: To learn to avoid giving in to first impressions, which can lead to misinterpretations. To find a middle ground between making hasty decisions, which has the risk of making bad decisions, and being too precise, which may lead to unnecessary loss of time. |
| **SESSION 3:**  **CHANGING BELIEFS** | Focus: Bias against disconfirmatory evidence, which is the tendency to cling to previous opinions, even when all the evidence indicates that you are wrong.  Aim: To explain to the group that it is often important to address the common tendency to cling to first impressions. This response bias favors incorrect decisions. It is therefore advisable to keep an open mind. |
| **SESSION 4:**  **EMPATHIZE (I)** | Focus: Theory of mind, perception of emotions.  Aim: To demonstrate that, although facial expressions are very important for understanding a person's state of mind and feelings, we can also easily misinterpret them. To interpret a facial expression properly it is important to consider other sources of information (*context; previous personal history…*). Participants learn to consider a variety of contextual information rather than relying on isolated details. |
| **SESSION 5:**  **MEMORY** | Focus: Overconfidence in errors.  Aim: To learn that memory is constructive and does not function as a recorder. One of the aims of this module is to teach the patients to question memories if a vivid recollection is not possible. In this case, more evidence is needed to remember properly, particularly for significant interpersonal situations (e.g., conflicts). |
| **SESSION 6:**  **EMPATHIZE (II)** | Focus: Theory of mind, need for closure on issues of concern, no tolerance of open-endedness or ambiguity.  Aim: To learn the difference between the information they may have and the information available in the exercises. |
| **SESSION 7:**  **JUMPING TO CONCLUSIONS (II)** | Focus: Jumping to conclusions bias.  Aim: To learn that it is essential to invest sufficient time in solving complex problems. Participants are shown several charts. Their task is to deduce the title of an illustration or picture from among four possible options. While for some pictures the solution is obvious, for others it only becomes clear after thorough consideration. |
| **SESSION 8:**  **MOOD** | Focus: Negative cognitive schemas.  Aim: To learn about cognitive biases that may contribute to the origin and maintenance of depression and low self-esteem. |

Figure 1 – Flowchart of the study


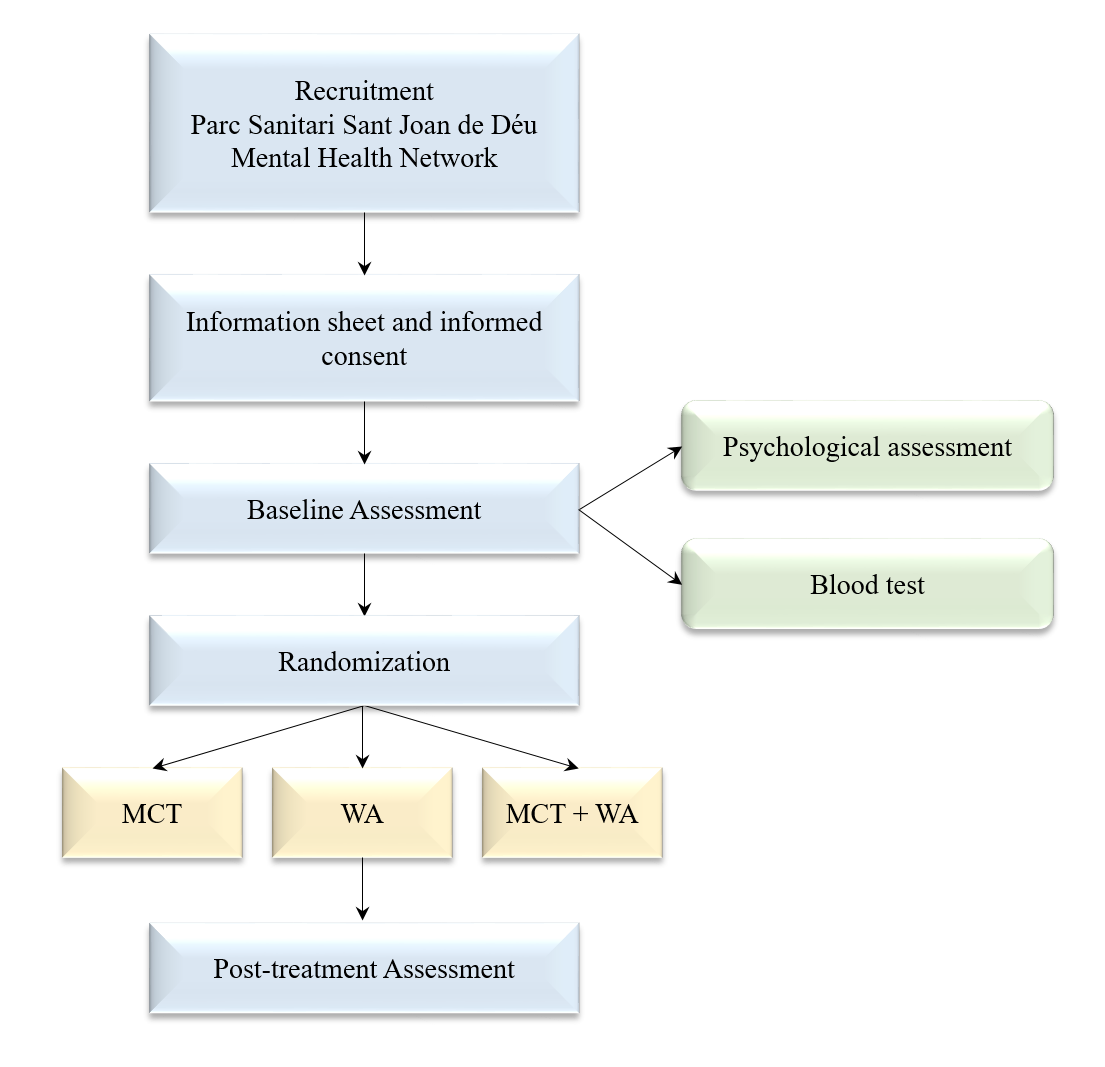

Supplement: Supplementary file 1 [file Table_1.DOCX]
